# *LmCYP4G102*: An oenocyte-specific cytochrome P450 gene required for cuticular waterproofing in the migratory locust, *Locusta migratoria*

**DOI:** 10.1038/srep29980

**Published:** 2016-07-22

**Authors:** Zhitao Yu, Xueyao Zhang, Yiwen Wang, Bernard Moussian, Kun Yan Zhu, Sheng Li, Enbo Ma, Jianzhen Zhang

**Affiliations:** 1 Institute of Applied Biology & School of Life Science, Shanxi University, Taiyuan, Shanxi 030006, China; 2 Robert-Bosch Krankenhaus, Institut für Klinische Pharmakologie, Auerbachstrasse 112, Stuttgart 70376, Germany; 3 Genetik der Tiere, Universität Tübingen, Auf der Morgenstelle 15, Tübingen 72076, Germany; 4 Angewandte Zoologie, TU Dresden, Zellescher Weg 20b, Dresden 01217, Germany; 5 iBV, Université Nice, Parc Valrose, Nice 06000, France; 6 Department of Entomology, 123 Waters Hall, Kansas State University, Manhattan, KS 66506, USA; 7 Key Laboratory of Insect Developmental and Evolutionary Biology, Institute of Plant Physiology and Ecology, Shanghai Institutes for Biological Sciences, Chinese Academy of Sciences, Shanghai 200032, China

## Abstract

Cytochrome P450 superfamily proteins play important roles in detoxification of xenobiotics and during physiological and developmental processes. To contribute to our understanding of this large gene family in insects, we have investigated the function of the cytochrome P450 gene *LmCYP4G102* in the migratory locust *Locusta migratoria*. Suppression of *LmCYP4G102* expression by RNA interference (RNAi) does not interfere with moulting but causes rapid loss of body weight - probably due to massive loss of water, and death soon after moulting. Accordingly, maintaining these animals at 90% relative humidity prevented lethality. Consistently, RNAi against *LmCYP4G102* provoked a decrease in the content of cuticular alkanes, which as an important fraction of cuticular hydrocarbons have been shown to confer desiccation resistance. In addition, the cuticle of *LmCYP4G102*-knockdown locusts was fragile and easier deformable than in control animals. Presumably, this phenotype is due to decreased amounts of cuticular water that is reported to modulate cuticle mechanics. Interestingly, *LmCYP4G102* was not expressed in the epidermis that produces the cuticle but in the sub-epdiermal hepatocyte-like oenocytes. Together, our results suggest that the oenocyte-specific *LmCYP4G102* plays a critical role in the synthesis of cuticular hydrocarbons, which are important for cuticle waterproofing and mechanical stability in *L. migratoria*

Cytochrome P450 monooxygenases (P450s or CYPs) belong to an ancient gene superfamily that is highly conserved in all organisms. These enzymes play an essential role in oxidation reactions in many important life processes^1^. CYP genes are abundant in insects; they generally participate in the metabolisms of plant secondary substances and pesticides and are also involved in insect growth and development by synthesizing endogenous substances, such as ecdysone, juvenile hormone (JH), fatty acids and cuticular hydrocarbons^2^,^3^,^4^,^5^.

Cytochrome P450 genes are expressed in a tissue-specific manner, indicating their diverse physiological functions^2^. Many CYPs that have been studied, especially in agricultural pest insects, were found to be involved in detoxification of insecticides. Among them, CYPs belonging to the CYP4, CYP6, CYP9 and CYP12 families are generally expressed in the midgut, Malpighian tubules and fat bodies^6^,^7^,^8^. CYPs expressed in the prothoracic glands are involved in hormone 20-hydroxyecdysone (20E) biosynthesis in *Drosophila melanogaster* and *Manduca sexta*^2^,^9^,^10^,^11^. However, *CYP15A1* is expressed selectively in the corpora allata of *Diploptera punctata*, which catalyses an epoxidation reaction in the JH biosynthesis pathway^4^. *CYP4G20* is expressed in the olfactory sensilla of *Mamestra brassicae*, suggesting its role in odorant and/or xenobiotic clearance^12^. Martins’ group previously investigated the transcriptome of oenocytes isolated from *Aedes aegypti* pupae and found that 8% of the oenocyte transcripts were coding cytochrome P450s^13^.

Oenocytes are derived from the ectoderm and are often large, polyploid cells. The location of oenocytes varies in different insect species. In *Drosophila*, oenocytes form subepidermal clusters located on both sides of each abdominal segment^14^; in *Apis mellifera*, oenocytes are present in groups below the epidermis in close association with the fat bodies^15^,^16^; and in *Tenebrio molitor*, oenocytes lie among the fat bodies and are tightly associated with the tracheal system^17^.

Although oenocytes were discovered more than 150 years ago, their function is still not completely understood. Several studies have indicated that insect oenocytes participate in detoxification processes and lipid metabolism^14^,^18^. In addition, CYPs expressed in oenocytes might participate in steroid metabolism^19^. However, the association between CYPs and ecdysone synthesis in oenocytes remains to be elucidated. Thus far, the only cytochrome P450 enzyme that has been detected in *Drosophila* oenocytes is CYP4G1, an oxidative decarbonylase for cuticular hydrocarbon biosynthesis^5^. Consistently, the role of oenocytes in the production of cuticular hydrocarbons has also been demonstrated in the German cockroach, *Blattella germanica*^20^.

The multiple functions of the CYP enzymes in insects are widely recognized, but there is limited knowledge on their physiological roles in oenocytes. In the present work, we identified an oenocyte-specific cytochrome P450 gene (*LmCYP4G102*) from *L. migratoria*. Suppression of *LmCYP4G102* expression by RNA interference (RNAi) led to rapid weight loss and dehydration-induced mortality. Consistently, the amounts of cuticular hydrocarbons (CHCs) that have been reported to constitute a waterproof barrier in insects^21^,^22^,^23^ were significantly reduced in ds*LmCYP4G102*-injected insects. In addition, their cuticle had lost resistance to mechanical stress. We believe, however, that this function of *LmCYP4G102* is indirect. Rather, consistent with the theory that water is a major modulator of cuticle stiffness^24^, massive water loss provoked by *LmCYP4G102* RNAi results in a dry and brittle cuticle.

## Results

### Molecular characterization of *LmCYP4G102*

**Figure 1:**
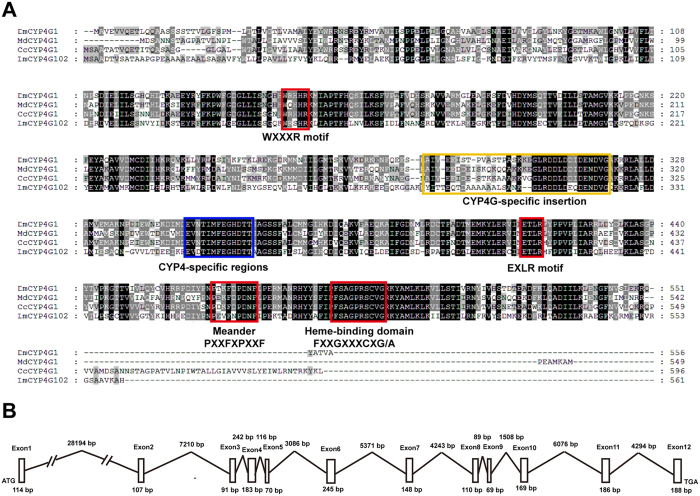
Multiple sequence alignments of the deduced CYP4G amino acid sequences in insects and gene structure analysis of *LmCYP4G102*. (**A**) The cytochrome P450 signature motifs (WXXXR, EXXR and PXXFXPXXF) and the heme-binding motif FXXGXXXCXG/A are boxed in red. The CYP4-specific regions and CYP4G-specific insertion are boxed in blue and yellow, respectively. Fully conserved amino acid sequences are boxed in black. *Musca domestica*, Md (CYP4G1, XP_005177793.1); *Drosophila melanogaster*, Dm (CYP4G1, AAF45503.1); *Ceratitis capitata*, Cc (CYP4G1, XP_004521059.1); *Locusta migratoria*, Lm (CYP4G102, KU833274). (**B**) Genomic organization of *LmCYP4G102*. The exon and intron sizes of *LmCYP4G102* are noted in the corresponding positions.

The predicted protein of *LmCYP4G102* from *L. migratoria* (GenBank accession number: KU833274) contains 561 amino acid residues and has a calculated molecular mass of 63.7 kDa. The deduced amino acid sequence has a heme-binding motif (FXXGXRXCXG/A) near the C-terminus, containing amino acid residues 491–500 and a cysteine residue, which is important for heme binding. The consensus sequence WXXXR is the structural element of Helix-C (heme-interacting region). A characteristic Asp/Glu-rich insertion of 34-residue sequence is unique to CYP4G P450s^5^. The conserved 13-residue motif EVDTIMFEGHDTT is a characteristic of the CYP family 4 and is not shared by other CYPs^25^. Furthermore, Helix-K (hydrogen-binding sequence) is shared among CYP genes with the consensus sequence EXXR. PXXFXPE/DRF is in the “meander” facing the EXLR motif. (Fig. 1A). Amino acid sequence alignments showed that LmCYP4G102 possessed characteristic motif sequences of CYP4Gs. The availability of a genomic sequence in the whole-genome shotgun sequencing project of *L. migratoria* allowed the determination of the exon/intron organization of *LmCYP4G102*, which includes 11 introns and 12 exons (Fig. 1B).

### Localization of oenocytes and the *LmCYP4G102* transcript

**Figure 2:**
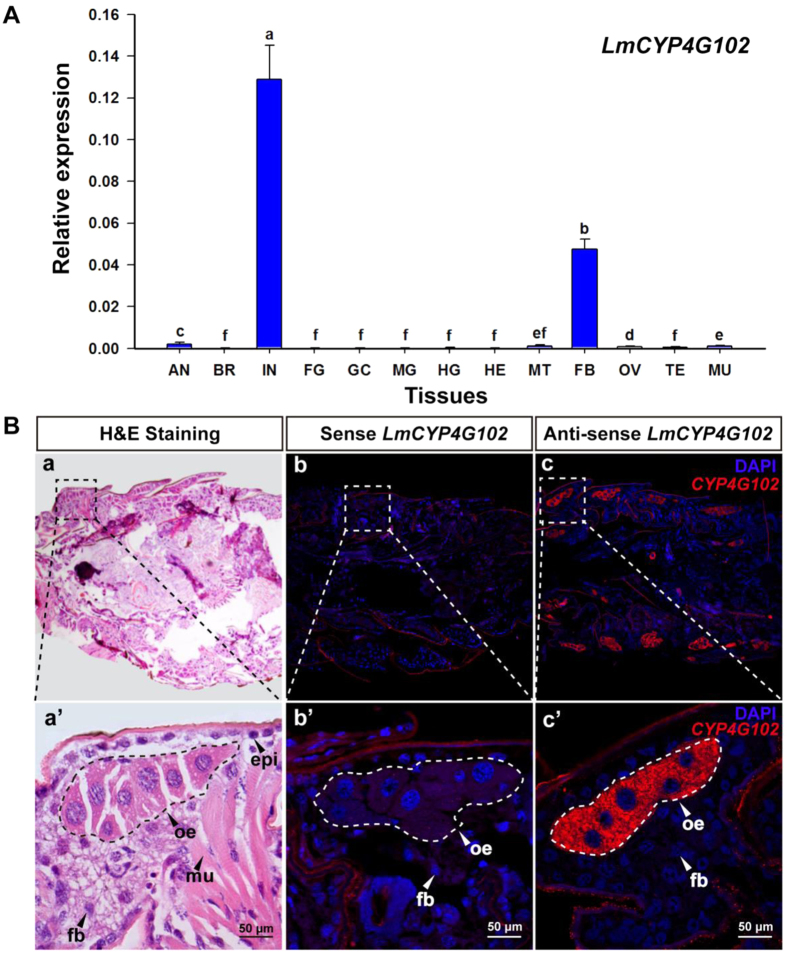
Tissue distribution and mRNA localization of *LmCYP4G102*. (**A**) Tissue-dependent expression of *LmCYP4G102* in fifth-instar nymphs, as determined by RT-qPCR. The tissues include antenna (AN), brain (BR), integument (IN), foregut (FG), midgut (MG), hindgut (HG), gastric caeca (GC), Malpighian tubules (MT), fat bodies (FB), muscles (MU), testis (TE), ovary (OV) and haemolymph (HE). *Rp49* was used as an internal reference gene. The relative expression of mRNA is indicated as the ratio of mRNA levels between *LmCYP4G102* and *Rp49*. Data are shown as means ± SE from three independent experiments. Different letters on the bars indicate significant difference among different tissues (*P* < 0.05, Tukey’s HSD test; n = 3). (**B**) The histological localization of oenocytes and *LmCYP4G102* in the first-instar nymphs of *L. migratoria*. The open squares indicate the magnifying position. (a) Location of oenocytes. Oenocytes appear as a group of cells located under the integument on both sides of each abdominal segment. (a’) The magnification of the square shown in (a). The clustered oenocytes are attached to the epidermis and are closely associated with the fat body; the boxed region shows one cluster. (b-b’, c-c’) Fluorescence *in situ* hybridization of *LmCYP4G102.* The mRNA of *LmCYP4G102* localized in oenocyte clusters. The boxed region shows one cluster. oe, oenocytes; epi, epidermal cell; fb, fat bodies; mu, muscle; tr, trachea. Blue, nuclear; red, *LmCYP4G102*. (Scale bars: 50 μm.)

To further study the function of *LmCYP4G102*, we determined its expression pattern and localization. The *LmCYP4G102* transcript was virtually expressed only in the integument and fat bodies, and no significant transcript levels were detected by RT-qPCR in the remaining 11 tissues tested, including antenna, brain, foregut, gastric caeca, midgut, hindgut, testis, ovary, muscle, Malpighian tubules and haemolymph, from fifth-instar nymphs of *L. migratoria* (Fig. 2A).

To determine the localization of *LmCYP4G102* mRNA in the integument of *L. migratoria*, we performed fluorescence *in situ* hybridization. A histological examination of the paraffin sections of the abdomen from the first-instar nymphs revealed that oenocytes were clustered underneath the abdominal epidermis and were closely associated with the fat bodies (Fig. 2B). We found that the *LmCYP4G102* transcript was predominately expressed in these oenocytes (Fig. 2B). These results consistently indicate that *LmCYP4G102* is specifically expressed in oenocytes that are associated with the fat bodies underneath the integument.

### Effect of LmCYP4G102 RNAi on locust nymphs

**Figure 3:**
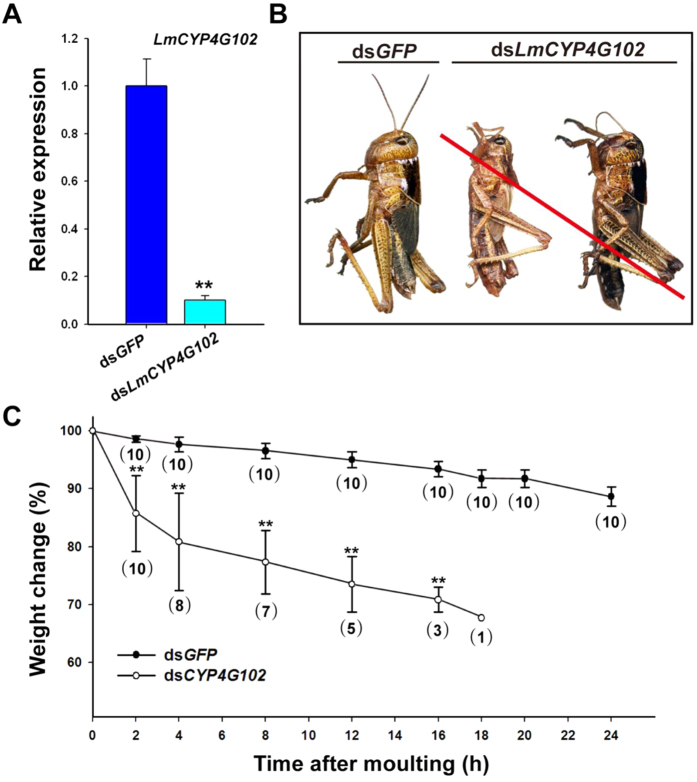
The phenotypes after the RNAi of *LmCYP4G102*. (**A**) The silencing efficiency of *LmCYP4G102.* The mRNA expression of *LmCYP4G102* was determined by RT-qPCR 24 h after dsRNA injection. *Rp49* was used as an internal reference gene. Data are shown as means ± SE from three independent experiments. The two asterisks ** indicate a significant difference between the ds*GFP*- and ds*LmCYP4G102*-injected locust groups (*P* < 0.01; Student’s *t*-test, n = 3). (**B**) The lethal phenotypes after the RNAi of *LmCYP4G102.* The ds*LmCYP4G102*-injected nymphs died shortly (<24 h) after moulting with 100% cumulative mortality (n = 40). The red slash line indicates that the insect has died with curly antennae and crumpled cuticle. (**C**) The body weight of ds*GFP*- and ds*LmCYP4G102*-injected locusts was measured from 0 to 24 h after moulting into fifth-instar nymphs (n = 10 each). The number in the parentheses indicates the number of nymphs that were measured; however, dead locusts were excluded from this evaluation. The results were calculated as the percentages of the body weight change, and the data are shown as means ± SE except for the ds*LmCYP4G102*-injected nymphs at 18 h (*P* < 0.01, Independent-Sample *t*-test).

To investigate the biological roles of *LmCYP4G102* in *L. migratoria*, we performed an RNA interference (RNAi) assay on newly moulted fourth-instar nymphs. To assess the efficiency of the RNAi, we verified the transcription level 24 h after dsRNA injection by using RT-qPCR. The injection of ds*LmCYP4G102* led to a significant decrease in the *LmCYP4G102* transcript (Fig. 3A). The suppression of *LmCYP4G102* had no effect on nymph development, and the nymphs could moult to the next stage; however, the newly moulted nymphs died shortly (<24 h) after moulting, with 100% cumulative mortality. The nymphs exhibited curly antennae and crumpled cuticle (Fig. 3B). Such ds*LmCYP4G102*-induced effects were essentially the same when the dsRNA was injected into second-, third-, fourth- and fifth-instar nymphs. When the fourth-instar nymphs were injected with ds*LmCYP4G102*, the body weight of the fifth-instar nymphs decreased by approximately 14.3% at 2 h after moulting, whereas the body weight decreased only 1.4% in ds*GFP*-injected nymphs (Fig. 3C).

**Figure 4:**
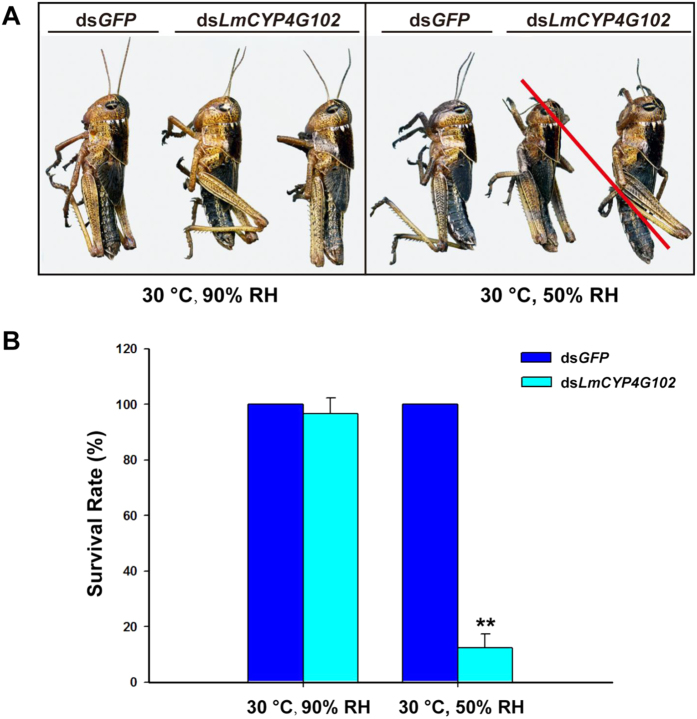
Effect of high humidity on the survival rate of the ds*LmCYP4G102*-injected nymphs. Fourth-instar nymphs injected with ds*GFP* or ds*LmCYP4G102* were transferred from 50% RH to 90% RH on day 7 before moulting (n = 32 each). (**A**) Maintaining ds*LmCYP4G102*-injected locusts at 90% RH prevented dehydration and death after moulting. However, when ds*LmCYP4G102*-injected locusts that had been maintained at 90% RH for 3 d were transferred to 50% RH, the nymphs dehydrated and died thereafter (red slash). No mortality was observed in the ds*GFP*-injected control group at 50% RH. (**B**) Survival rates of the ds*GFP*- and ds*LmCYP4G102*-injected fifth-instar nymphs under different humidity conditions at 24 hr. 3.1 and 87.6% of the ds*LmCYP4G102*-injected nymphs died at 90% RH and 50% RH, respectively. However, no mortality was observed in the ds*GFP*-injected control group under either RH condition. Data are shown as means ± SE from three independent experiments. The two asterisks ** indicate a significant difference between the ds*GFP*- and ds*LmCYP4G102*-injected locust groups (*P* < 0.01; Student’s *t*-test, n = 3).

These results suggest that lethality caused by ds*LmCYP4G102* injection is due to loss of water. To test this possibility we asked whether high humidity would rescue lethality. Fourth-instar locusts that had been injected with ds*LmCYP4G102* were transferred from 50% relative humidity (RH) to 90% RH on day 7 before moulting. The ds*LmCYP4G102*-injected locusts could moult to the next stage and developed normally at 90% RH (Fig. 4A). After three days at 90% RH, we transferred them to an atmosphere with 50% RH. However, unlike ds*LmCYP4G102*-injected control locusts at 90% RH and ds*GFP*-injected control locusts at 50% RH, approximately 87.6% humidity-rescued ds*LmCYP4G102* locusts became dehydrated and died after 24 h at 50% RH (Fig. 4).

**Figure 5:**
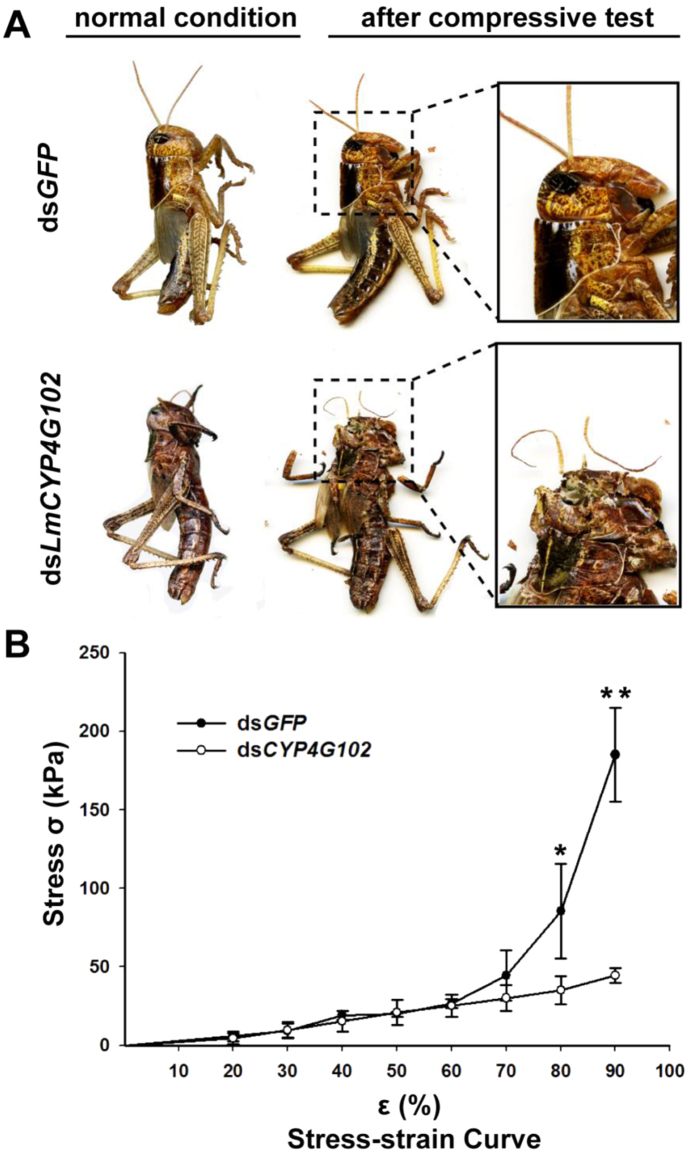
Biomechanics of nymphs after the RNAi of *LmCYP4G102.* (**A**) To understand the impact of *LmCYP4G102* on cuticle mechanics, we analysed the phenotype of locusts in a compression test. The bodies that had been injected with ds*LmCYP4G102* broke apart after mechanical challenge, while control insects showed an intact body. (**B**) Comparison of the stress-strain curves in ds*GFP* and ds*LmCYP4G102* groups of insects. Locusts were compressed with a 50-N load cell and a constant displacement rate of 5 mm/min. The stress of both curves linearly increases with increasing strain. Suppression of *LmCYP4G102* expression resulted in significantly decreased stress resistance compared to control nymphs when the strain reached to 80% and 90%. The data are shown as means ± SE. The asterisks * and ** above the bars indicate significant differences at *P* < 0.05 and 0.01, respectively, between the ds*GFP*- and ds*LmCYP4G102*-injected locust groups (Student’s *t*-test, n = 8).

The dead nymphs that had been injected with ds*LmCYP4G102* were brittle and fragile in response to pressure (Fig. 5A). This observation suggests that the mechanical properties of the cuticle may depend on *LmCYP4G102* function. Our compression tests confirmed that ds*LmCYP4G102*-injected insects required less strain for deformation than did ds*GFP*-injected insects (Fig. 5B). Our results suggest that stress resistance of the cuticle of ds*LmCYP4G102*-injected insects was significantly reduced compared to that of the control insects that were injected with ds*GFP*.

### Analysis of cuticular hydrocarbons (CHCs)

**Figure 6:**
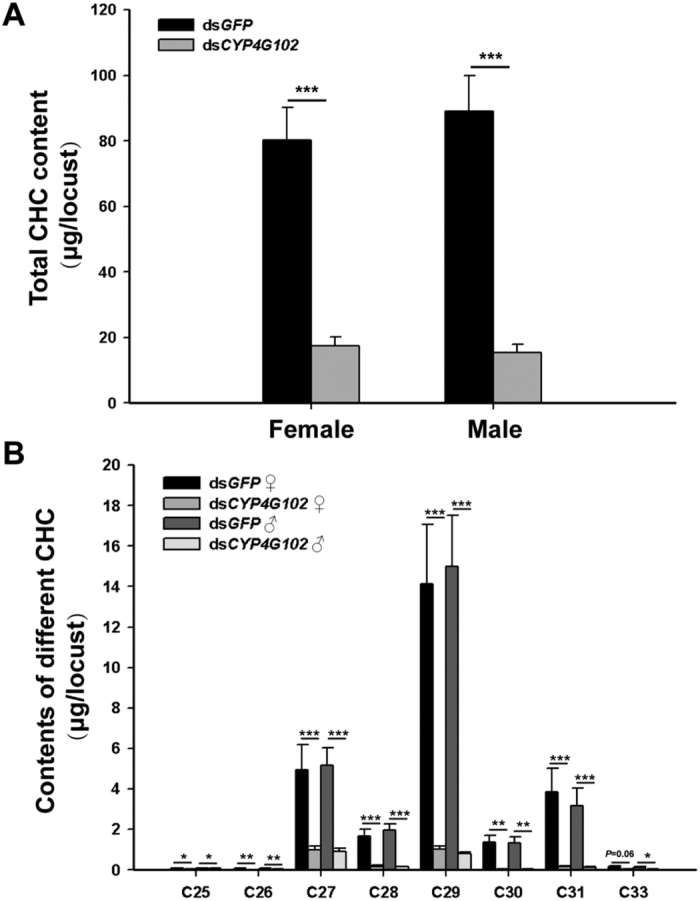
Effect of *LmCYP4G102* RNAi on the cuticular hydrocarbon contents of *L. migratoria*. (**A**) Total hydrocarbon contents extracted from female and male locusts. (**B**) Contents of straight-chain alkanes of different lengths (C25–C33). The hydrocarbons are expressed as micrograms (μg) per locust, and the data are shown as means ± SE. The hydrocarbons content was significantly different in the ds*LmCYP4G102*-injected group compared to those in the ds*GFP*-injected group. The asterisks *, ** and *** above the bars indicate significant differences at *P *< 0.05, 0.01 and 0.001, respectively, between the ds*GFP*- and ds*LmCYP4G102*-injected locust groups for the same sex (Student’s *t*-test, n = 12-13).

Our GC-MS analyses of CHCs showed that ds*GFP*-injected locusts had a mixture of hydrocarbons that primarily corresponded to alkanes (lacking of alkenes), including n-alkanes and branched alkanes. The major branched alkanes included 3-methylalkanes; mixtures of 9-, 13-, 15-, and 17-methylalkanes; and dimethylalkane. N-nonacosane (C29) was present in large quantities (Supplementary Table S1). The total alkane content differed significantly between the ds*LmCYP4G102*- and ds*GFP*- injected groups but did not differ between males and females (Fig. 6A). The mean values of total alkanes per locust were 80.30 ± 9.85 μg and 17.53 ± 2.69 μg for ds*GFP*- and ds*LmCYP4G102*-injected females, respectively, and 88.95 ± 11.05 μg and 15.37 ± 2.58 μg for ds*GFP*- and ds*LmCYP4G102*-injected males, respectively. These results showed significant differences between the ds*GFP*- and ds*LmCYP4G102*-injected insects in both females and males. However, the number of alkane peaks was not noticeably different between the ds*GFP*-injected and ds*LmCYP4G102*-injected locusts. Apparently, such a dramatic decrease in ds*LmCYP4G102*-injected locusts was due to decreases in all different alkanes, largely due to decreased n-nonacosane (C29) contents (Fig. 6B and Supplementary Table S1).

## Discussion

Cytochrome P450s have essential physiological functions and play a major role in the adaptation of insects to their environment^1^. In the present work, we identified an oenocyte-specific cytochrome P450 gene of the CYP4 family, *LmCYP4G102*, in the migratory locust *Locusta migratoria* that is needed for cuticle barrier function and stability.

### LmCYP4G102 belongs to the CYP4 family of cytochrome P450 proteins and is expressed in the oenocytes

The CYP4 family is one of the most ancient and diversified CYP groups^1^. The LmCYP4G102 protein sequence exhibits a high similarity to members of the subfamily 4G, which represent very few CYP orthologues distributed across Insecta^5^. Although specialized roles of some CYP4G subfamily enzymes have been reported, the physiological roles of many of these enzymes remain unknown or theoretical. For example, *CYP4G8* and *CYP4G19* are overexpressed in pyrethroid-resistant strains of *Helicoverpa armigera*^26^ and *Blattella germanica*^27^, respectively, suggesting their possible involvement in pyrethroid resistance. A study on *CYP4G15* in the fruit fly *D. melanogaster* demonstrated its expression in the central nervous system, which suggests its role in metabolism of endogenous compounds rather than the detoxification of xenobiotics^28^. In the cabbage armyworm *M. brassicae*, the expression of *CYP4G20* in antennae may be related to its metabolic role as various odorants and xenobiotics enter the olfactory sensillae from the external environment^12^. In *Antheraea yamamai*, *CYP4G25* was found to be expressed in the integument during insect diapause; therefore, its role was hypothesized to be in the diapause termination pathway^29^. In *D. melanogaster*, *CYP4G1* is the only CYP gene that has been detected in oenocytes so far and is known for its role in the synthesis of CHCs^5^. Our *in situ* hybridization data showed that *LmCYP4G102* was specifically expressed in oenocytes in *L. migratoria*. Although our tissue-dependent expression profiles of*LmCYP4G102* demonstrated its predominant expression in the integument and the fat bodies of *L. migratoria* (Fig. 2A), we think that this expression pattern is due to the strong association of oenocytes with the integument and the fat bodies.

### LmCYP4G102 is involved in production of cuticular hydrocarbons

A layer of lipids coating the cuticular surface is considered as an important constituent of the waterproof barrier to control cuticular water loss in insects^21^,^22^,^23^. Cuticular hydrocarbons (CHCs) have been demonstrated to be the main components of this layer in many species^30^,^31^. Hydrocarbons comprise 37.6 and 50.7% of cuticular lipids extracted from the adult body of male and female *L. migratoria*, respectively^30^. RNAi against *CYP4G102* provoked reduced levels of total CHCs, largely due to decreased n-nonacosane (C29) contents. In *D. melanogaster*, the RNAi knockdown of *CYP4G1* in oenocytes resulted in the elimination of almost all of the CHCs. Moreover, this gene has been functionally characterized in heterologous expression systems, showing that CYP4G1 is an oxidative decarboxylase that converts long-chain aldehydes into hydrocarbons^5^. Because CHCs biosynthesis takes place in oenocytes^20^, those genes that are expressed in these cells are candidates for their involvement in CHC synthesis. In summary, our data demonstrate that the oenocyte-specific cytochrome P450 gene*, LmCYP4G102*, which is homologous to *D. melanogaster CYP4G1*, participates at the production of cuticular alkanes in *L. migratoria*. The exact biochemical function of LmCYP4G102 remains to be investigated.

### LmCYP4G102 is involved in the formation of a barrier against water loss

Maintaining water balance is critical for insect survival in terrestrial environments. Our data demonstrate that locusts that were injected with ds*LmCYP4G102* died within a few hours after moulting when they were maintained at 30 °C and 50% RH, which are standard rearing conditions for locusts. These insects lost body weight quickly, much faster than did the control locusts that were injected with ds*GFP* (Fig. 3C). Rapid loss of body weight is conceivably due to rapid water loss. This defect could be countered by maintaining the *dsLmCYP4G102*-injected locusts at high humidity (90%) (Fig. 4). Taken together, *LmCYP4G102* is needed to establish a barrier against uncontrolled water loss. Most probably, this barrier function relies on an intact cuticle.

### Cuticle stiffness depends on LmCYP4G102

The mechanical properties of the cuticle depend to a large extent on the presence of water^24^. When we used a compression test to examine the mechanical properties of the cuticles of dehydrated and dead nymphs after injection with ds*LmCYP4G102*, we found that the respective locusts could easily break apart after mechanical challenge. Compared to the control nymphs that were injected with ds*GFP*, the nymphs that were injected with ds*LmCYP4G102* could withstand much less mechanical force (Fig. 5B). It is well known that the mechanical properties, i.e. stiffness and elasticity of the insect cuticle are modulated by the water content^32^,^33^,^34^,^35^. Accordingly, previous studies indicated that the endocuticle of locusts becomes harder by a factor of up to 9 and stiffer by a factor of up to 7.4 after drying^24^. Based on our data, we propose that massive water loss in ds*LmCYP4G102*-injected locusts results in an increased fragility of the cuticle. Thus, *CYP4G102* in *L. migratoria* seems to play a critical albeit indirect role in resistance of the cuticle against mechanical stress.

## Conclusion

We identified and characterized an oenocyte-specific cytochrome P450 gene, *LmCYP4G102*, from *L. migratoria*. Several lines of evidence - including a dramatic decrease in total cuticular alkane content, a rapid decrease of body weight probably due to rapid water loss, a significant increase in cuticular brittleness in ds*LmCYP4G102*-injected locusts - support our conclusion that *LmCYP4G102* plays a critical role in cuticular waterproofing and stability and is therefore essential for insect survival. In summary, LmCYP4G102 seems to have a similar function as the oenocyte-specific CYP protein DmCYP4G1, a well-characterized enzyme implied in the biosynthesis of cuticular hydrocarbons in *D. melanogaster*. This work, hence, underlines the importance of the oenocytes in cuticle formation and function.

## Materials and Methods

### Insect Rearing

*L. migratoria* were purchased from Insect Protein Co., Ltd., Cangzhou City, China, and reared in a growth chamber at 30 ± 2 °C and 40 ± 10% relative humidity (RH). The locusts were fed fresh wheat sprouts under a 14:10-h light:dark photoperiod. For the high humidity rescue experiments, fourth-instar nymphs injected with dsRNA were transferred to a humidity chamber (90%RH) at 30 °C on day 7 before moulting.

### Sequencing of cDNA encoding CYPs

CYP-like genes from *L. migratoria* were obtained by searching the locust transcriptome database. A cDNA sequence putatively encoding an enzyme of the CYP4 family was selected for subsequent work. The translated amino acid sequence was searched using BLASTP in the NCBI database (http://www.ncbi.nlm.nih.gov/) to further reveal its identity. To sequence the full-length cDNA of the CYP gene, M-MLV Reverse Transcriptase (TaKaRa, Japan) and an oligo-(dT)_18_ primer (TaKaRa, Japan) were used to synthesize the first-strand cDNA with 1 μg of total RNA that was extracted from the whole body of fifth-instar nymphs by RNAiso Plus (TaKaRa, Japan). PCR was performed with the synthetic cDNA and gene-specific primers (Table 1). Specific fragments were purified with a gel purification kit (Omega, USA), cloned into the pEASY-T3 vector (TransGen, China), and completely sequenced from both directions.

### Deduced amino acid sequences and gene structure analysis of *LmCYP4G102*

The prediction of the open reading frame (ORF) and the translation of the cDNA sequence into amino acid sequence were performed using the Translate tool on the ExPASy Proteomics web site (http://web.expasy.org/translate/). The locust CYP gene was finally named CYP4G102 by the P450 Gene Family Nomenclature Committee (Dr. D.R. Nelson, University of Tennessee, Memphis).

To compare the amino acid sequences and conserved domains, sequence alignments of the deduced amino acid sequences of insect CYP4Gs were performed using GeneDoc software. The conserved motifs of CYPs were identified based on references previously described by Feyereisen^1^. The molecular weight (Mw) were predicted using the Compute pI/Mw tool (http://web.expasy.org/compute pi/). To reveal the genomic structure of *LmCYP4G102*, BLASTN was used to search the exons and introns in the *L. migratoria* assembly genome database (GenBank: GCA_000516895.1). The GT-AG rule was used to distinguish exon-intron boundaries throughout the whole process.

### Tissue-dependent expression of *LmCYP4G102*

To analyse tissue expression profiles, different tissues, including antenna, brain, integument, foregut, gastric caeca, midgut, hindgut, fat bodies, testis, ovary, muscle, Malpighian tubules and haemolymph, were dissected from 3-day-old fifth-instar nymphs of *L. migratoria*. Total RNA was isolated with three replications using RNAiso plus (TaKaRa, Japan) according to the manufacturer’s instructions. The extracted RNA was treated with RNase-free DNaseI (Takara, Japan) to remove genomic DNA. The quality and quantity of RNA were verified on a 1% agarose gel and with a NanoDrop 2000 spectrophotometer (Thermo Fisher Scientific, USA), respectively.

One microgram of total RNA was used to synthesize the first-strand cDNA with M-MLV Reverse Transcriptase (Takara, Japan). The tissue-dependent expression profiles of *LmCYP4G102* were measured by reverse transcription quantitative PCR (RT-qPCR). *Rp49* was used as a reference gene for normalization^36^. The *LmCYP*4*G102*- and *Rp49*-specific primers that were used for the RT-qPCR analysis are shown in Table 1. RT-qPCR was performed using the SYBR Green qPCR Master Mix (TOYOBO, Japan) on an ABI 7300 detection system (ABI, USA). The cycling conditions were 1 min at 95 °C and 40 cycles of 15 s at 95 °C and 31 s at 60 °C. The melting curve was evaluated for each RT-qPCR experiment to confirm the amplification specificity. The PCR reactions were performed in three biological replicates, with a technical duplicate for each RNA sample. The relative expression was determined using the 2^−ΔΔCt^ method of relative quantification^37^.

### Fluorescence *in situ* hybridization

Digoxigenin (DIG)-labelled antisense and sense probes of *LmCYP4G102* were generated from linearized recombinant pGEM-T plasmids using the Dig RNA-Labelling Kit (Roche, Switzerland) according to the manufacturer’s instructions.

The primers that were used for the probe synthesis of *LmCYP4G102* are listed in Table 1. The abdomens of first-instar nymphs were fixed in 4% (wt/vol) paraformaldehyde at 4 °C overnight. The samples were embedded in paraffin by a conventional method. Sections (5 μm) were prepared and mounted onto SuperFrost Plus slides and used for haematoxylin and eosin (H & E) staining and fluorescence *in situ* hybridization. For fluorescence *in situ* hybridization, the paraffin-embedded tissue slides were deparaffinized in xylene and rehydrated with an ethanol gradient. After digestion with 20 μg/mL proteinase K (Roche, Switzerland) at 37 °C for 30 min, the tissues were prehybridized with prehybridization solution (Boster, China). The slides were then hybridized with *LmCYP4G102* probes (DIG-labelled antisense or sense RNA) in a humid box at 37 °C overnight. After hybridization, the slides were successively washed in 2 × SSC, 1 × SSC, and 0.2 × SSC at 37 °C and then incubated in anti-digoxigenin-alkaline phosphatase conjugate (1:150 dilution) for 30 min at 37 °C for probe detection. The fluorescent signal of digoxigenin (DIG) was obtained by HNPP/Fast Red (Roche, Switzerland). Then, the nuclei were stained with DAPI for 10 min at room temperature. Images were captured on an LSM 710 confocal fluorescence microscope (Zeiss, Germany) at magnifications of 20X and 63X.

### RNA interference analysis of *LmCYP4G102*

Double-stranded RNA (dsRNA) was synthesized as previously described^38^. The specific primers with the T7 RNA polymerase promoter sequence at the 5′-end that were used for ds*GFP* and ds*LmCYP4G102* synthesis are shown in Table 1. The synthesized dsRNA was dissolved in nuclease-free water, and the final concentration of dsRNA was adjusted to 1.5 μg/μl. Aliquots of 6 μg (4 μl) of dsRNA of *LmCYP4G102* or *GFP* were injected into the abdomen (between the second and third abdominal segments) of the newly moulted fourth-instar nymphs using a manual microsyringe (Ningbo, China). ds*GFP*-injected nymphs were used as negative controls. Each group consisted of 50 individual nymphs, and the experiment was carried out in three replicates. To analyse the suppression level of *LmCYP4G102* transcripts, cDNAs were synthesized from total RNA that had been isolated from whole nymphs 24 h after dsRNA injections. The total RNA was independently isolated for each of the three replications.

The body weight of ds*GFP*- and ds*LmCYP4G102*-injected nymphs (n = 10 each group) was measured from 0 to 24 h after moulting into fifth-instar nymphs. However, dead locusts were excluded from this evaluation. The results were calculated as the percentages of the body weight change, and data are shown as means ± SE.

### Uniaxial compression test

A uniaxial compression test was used to determine the mechanical behaviour of dead nymphs that were injected with ds*LmCYP4G102* or ds*GFP*. Treated and control insects were maintained under the same conditions (30 °C, 50% RH). The insects were compressed between two metal plates with a 50-N load cell and a constant displacement rate of 5 mm/min using Instron 5544 tester (Instron, USA). A computer was used to record the displacement and load signals throughout the entire loading process. For the stress-strain curves of specimens, the compressive stress was the ratio of the applied load (in N) to the original specimen area, whereas the compressive strain was the ratio of the deformation to the specimen.

### Extraction and quantification of cuticular hydrocarbons (CHCs)

Aliquots of 3 μg of *LmCYP4G102* dsRNA were injected into 2-day-old second-instar nymphs. The surviving nymphs were collected after moulting to the next stage. Each nymph was identified by sex, weighed, immersed in 0.5 mL of hexane, and agitated gently for 2 min, after which the solvent was decanted into a clean vial. This procedure was repeated two more times. The three hexane extracts were combined and subjected to CHC purification and analysis by gas chromatography-mass spectrometry (GC-MS) on a TRACE 1310 coupled to an ISQ single-quadrupole MS detector with Xcalibur 2.2 software (Thermo Fisher Scientific, USA). The splitless injection of 1 μL was made into a HP-5MS UI capillary column (30 m × 0.32 mm × 0.25 μm). The carrier gas was helium with a flow of 1 mL/min. The initial temperature was held at 60 °C for 2 min, increased to 200 °C at a rate of 30 °C/min, and then increased to 320 °C and held for 10 min. Some of the alkanes were identified by their retention times compared to those of known standards (C7-C40 saturated alkanes Std, SUPELCO). The remaining alkanes were identified by Kovats retention index (*I*), which was calculated by an equation using retention time. These alkanes were quantified by their peak areas compared to that of the internal standard.

### Statistical analysis

All of the statistical analyses were performed using the SPSS program (SPSS Inc., USA). The *LmCYP4G102* relative expression levels were subjected to ANOVA followed by Tukey’s honestly significant difference (HSD) test to separate the mean values among tissues. The silencing efficiency of *LmCYP4G102*, the uniaxial compression results and the comparisons of the cuticular hydrocarbons between ds*LmCYP4G102* treatments and ds*GFP* controls were analysed using Student’s *t*-test. The comparison in body weight changes between the ds*LmCYP4G102*- and ds*GFP*-injected insects were analysed using Independent-sample *t*-test. *P* < 0.05 was set as significant for the comparisons.

## Additional Information

**How to cite this article**: Yu, Z. *et al*. *LmCYP4G102:* An oenocyte-specific cytochrome P450 gene required for cuticular waterproofing in the migratory locust, *Locusta migratoria*. *Sci. Rep.* **6**, 29980; doi: 10.1038/srep29980 (2016).

## Supplementary Material

Supplementary Information

## Figures and Tables

**Table 1:** Primers used for PCR amplification, *in situ* hybridization and dsRNA synthesis.

Application of primers	Primer names	Sequence of primers (5′–3′)	Products (bp)
Full-length cDNA amplification	*CYP4G102*-F	CAGCAGCAGCAGCAAGCATGT	1896
	*CYP4G102*-R	GGTGCGTTAACAGTGCCGATTAC	
RT-qPCR analysis	*CYP4G102*-F	GGGCATCCATCAGGACATTC	156
	*CYP4G102*-R	ATAGGCACAGGCGGGAACAT	
	*rp49-F*	CGCTACAAGAAGCTTAAGAGGTCAT	66
	*rp49*-R	CCTACGGCGCACTCTGTTG	
*in situ* hybridization	*CYP4G102*-F	TGGGCACCTACAAGATCCAC	458
	*CYP4G102*-R	GACCTGCATAATGAGAAACGT	
dsRNA synthesis	ds*CYP4G102*-F	taatacgactcactatagggTGGGCACCTACAAGATCCAC	498
	ds*CYP4G102*-R	taatacgactcactatagggGACCTGCATAATGAGAAACGT	
	ds*GFP*-F	taatacgactcactatagggGTGGAGAGGGTGAAGG	571
	ds*GFP*-R	taatacgactcactatagggGGGCAGATTGTGTGGAC	
