# Supplementary material for: LmCYP4G102: An oenocyte-specific cytochrome P450 gene required for cuticular waterproofing in the migratory locust, Locusta migratoria
Source: Sci Rep. 2016 Jul 22;6:29980. doi: 10.1038/srep29980 (PMC4957221; doi:10.1038/srep29980)
Supplement: Supplementary Information [file srep29980-s1.doc]

***LmCYP4G102*: An oenocyte-speciﬁc cytochrome P450 gene required for cuticular waterproofing in the migratory locust, *Locusta migratoria***

Zhitao Yu ^a^**^⊙^**, Xueyao Zhang ^a^**^⊙^**, Yiwen Wang ^b^, Bernard Moussian ^c^, Kun Yan Zhu ^d^, Sheng Li ^e^, Enbo Ma ^a^* and Jianzhen Zhang ^a^ *

^a^ Institute of Applied Biology & College of Life Science, Shanxi University, Taiyuan, Shanxi 030006, China

^b^ Genetik der Tiere, Universität Tübingen, Auf der Morgenstelle 15, Tübingen, Germany

^c^ Angewandte Zoologie, TU Dresden, Zellescher Weg 20b, Dresden, Germany and iBV, Université Nice, Parc Valrose, 06000 Nice, France

^d^ Department of Entomology, 123 Waters Hall, Kansas State University, Manhattan, KS 66506, USA

^e^ Key Laboratory of Insect Developmental and Evolutionary Biology, Institute of Plant Physiology and Ecology, Shanghai Institutes for Biological Sciences, Chinese Academy of Sciences, Shanghai 200032, China

* Author for correspondence ([maenbo2003@sxu.edu.cn](mailto:maenbo2003@sxu.edu.cn); [zjz@sxu.edu.cn](mailto:zjz@sxu.edu.cn))

⊙ These authors contributed equally to this work.

**Supplemental Data**

**Table S1. Cuticular hydrocarbons (CHC) profiles of *L. migratoria* after RNAi.**

| CHCs | kovats retention index (*I*) | *GFP* RNAi  female (n=6) | *GFP* RNAi  male (n=6) | *LmCYP4G102* RNAi  female (n=7) | *LmCYP*4G102 RNAi  male (n=6) |
| --- | --- | --- | --- | --- | --- |
| Total CHCs |  | 80.30 ± 9.85 | 88.95 ± 11.05 | 17.53 ± 2.69 | 15.37 ± 2.58 |
| **C25** | 2500 | 0.09 ± 0.02 | 0.09 ± 0.02 | 0.04 ± 0.01 | 0.05 ± 0.02 |
| 9-;11-;13-MeC25 | 2534 | 0.16 ± 0.05 | 0.19 ± 0.05 | 0.08 ± 0.02 | 0.09 ± 0.04 |
| 3-MeC25 | 2574 | 0.06 ± 0.03 | 0.05 ± 0.01 | 0.04 ± 0.02 | 0.05 ± 0.02 |
| 5,13-DimeC25 | 2583 | 0.01 ± 0.00 | 0.02 ± 0.00 | 0.01 ± 0.00 | 0.01 ± 0.00 |
| **C26** | 2600 | 0.06 ± 0.01 | 0.06 ± 0.01 | 0.02 ± 0.00 | 0.02 ± 0.01 |
| 11-; 13-MeC26 | 2633 | 0.06 ± 0.01 | 0.07 ± 0.02 | 0.03 ± 0.01 | 0.04 ± 0.02 |
| 4-MeC26 | 2658 | 0.01 ± 0.01 | 0.02 ± 0.01 | 0.01 ± 0.00 | 0.01 ± 0.00 |
| 3-MeC26 | 2674 | 0.08 ± 0.02 | 0.10 ± 0.02 | 0.03 ± 0.01 | 0.04 ± 0.02 |
| **C27** | 2700 | 4.93 ± 1.27 | 5.18 ± 0.84 | 1.00 ± 0.17 | 0.91 ± 0.16 |
| 9-;11-;13-MeC27 | 2733 | 1.73 ± 0.35 | 1.25 ± 0.55 | 0.80 ± 0.16 | 0.69 ± 0.19 |
| 3-MeC27 | 2774 | 6.08 ± 1.17 | 8.00 ± 1.29 | 2.52 ± 0.36 | 2.29 ± 0.40 |
| **C28** | 2800 | 1.66 ± 0.36 | 1.98 ± 0.29 | 0.18 ± 0 .04 | 0.15 ± 0.02 |
| **C29** | 2900 | 14.14 ± 2.93 | 14.98 ± 2.53 | 1.01 ± 0.16 | 0.78 ± 0.10 |
| 9-;11-;13-MeC29 | 2932 | 7.46 ± 1.60 | 9.17 ± 2.07 | 2.49 ± 0.32 | 2.12 ± 0.33 |
| 3-MeC29 | 2974 | 6.91 ± 1.35 | 7.35 ± 1.17 | 1.06 ± 0.14 | 0.82 ± 0.13 |
| **C30** | 3000 | 1.35 ± 0.35 | 1.31 ± 0.33 | 0.05 ± 0.01 | 0.05 ± 0.01 |
| 12-MeC30 | 3032 | 0.89 ± 0.10 | 1.05 ± 0.16 | 0.18 ± 0.03 | 0.16 ± 0.03 |
| **C31** | 3100 | 3.84 ± 1.18 | 3.18 ± 0.84 | 0.17 ± 0.03 | 0.14 ± 0.03 |
| 11-; 13-MeC31 | 3133 | 10.29 ± 1.19 | 11.43 ± 1.48 | 1.97 ± 0.31 | 1.67 ± 0.25 |
| 11,15-;11,17-;11,9-DimeC31 | 3162 | 0.98 ± 0.10 | 1.09 ± 0.20 | 0.30 ± 0.05 | 0.26 ± 0.04 |
| 3-MeC31 | 3175 | 0.46 ± 0.17 | 0.41 ± 0.12 | 0.02 ± 0.00 | 0.02 ± 0.00 |
| 12-;13-;14-MeC32 | 3232 | 0.51 ± 0.07 | 0.61 ± 0.08 | 0.07 ± 0.01 | 0.07 ± 0.02 |
| **C33** | 3300 | 0.16 ± 0.05 | 0.12 ± 0.04 | 0.05 ± 0.01 | 0.04 ± 0.01 |
| 11-;13-;15-MeC33 | 3330 | 3.42 ± 0.47 | 4.06 ± 0.51 | 0.55 ± 0.09 | 0.45 ± 0.09 |
| 11,15-;13,17-;15,19-DimeC33 | 3358 | 3.18 ± 0.45 | 3.43 ± 0.38 | 0.96 ± 0.15 | 0.90 ± 0.15 |
| 10-;12-;13-;14-MeC34 | 3430 | 0.20 ± 0.03 | 0.23 ± 0.03 | 0.03 ± 0.01 | 0.03 ± 0.01 |
| 12,20-;12,22-;13,21-DimeC34 | 3457 | 0.99 ± 0.15 | 1.16 ± 0.14 | 0.27 ± 0.05 | 0.24 ± 0.05 |
| 9-; 11-; 13-; 15-MeC35 | 3530 | 0.74 ± 0.11 | 0.83 ± 0.12 | 0.12 ± 0.02 | 0.09 ± 0.02 |
| 11,15-;13,17-;15,19-DimeC35 | 3558 | 9.17 ± 0.84 | 9.96 ± 1.00 | 3.22 ± 0.61 | 2.88 ± 0.59 |
| x,x-DimeC36* | 3657 | 0.15 ± 0.02 | 0.16 ± 0.02 | 0.05 ± 0.01 | 0.05 ± 0.01 |
| 11,15-;13,17-;15,19-DimeC37 | 3756 | 0.54 ± 0.09 | 0.53 ± 0.07 | 0.23 ± 0.05 | 0.28 ± 0.10 |

ds*GFP* or ds*LmCYP4G102* were injected into 2-day-old second-instar nymphs. The surviving nymphs were collected after moulting to the next stage, and cuticular hydrocarbons measured by a GC-MS proﬁle of hexane extract. CHC identities and their corresponding Kovats retention index (*I*) are given in the ﬁrst and second column, respectively. Each compound of hydrocarbons are shown as micrograms (μg) per locust and the data are shown as the mean ± SE (n=12-13) in each group. The hydrocarbons content was signiﬁcantly different in the ds*CYP4G102*-injected group compared to those in the ds*GFP*-injected group (*P*< 0.001, Student’s *t*-test). For dimethylalkane marked with an asterisk (*), the position of the methyl bond is not precisely known.
